# Nutraceutical Targeting of Inflammation-Modulating microRNAs in Severe Forms of COVID-19: A Novel Approach to Prevent the Cytokine Storm

**DOI:** 10.3389/fphar.2020.602999

**Published:** 2020-12-11

**Authors:** Michel Desjarlais, Maëlle Wirth, Isabelle Lahaie, Pakiza Ruknudin, Pierre Hardy, Alain Rivard, Sylvain Chemtob

**Affiliations:** ^1^ Department of Ophthalmology, Maisonneuve-Rosemont Hospital Research Center, Université de Montréal, Montréal, QC, Canada; ^2^ Departments of Pediatrics, Ophthalmology and Pharmacology, Centre Hospitalier Universitaire Sainte-Justine Research Center, Montréal, QC, Canada; ^3^ Department of Medicine, Centre Hospitalier de l'Université de Montréal (CHUM) Research Center, Montréal, QC, Canada

**Keywords:** COVID-19, microRNA, inflammation, cytokine storm, nutraceutics

## Abstract

The coronavirus disease 2019 (COVID-19) pandemic has become the number one health problem worldwide. As of August 2020, it has affected more than 18 million humans and caused over 700,000 deaths worldwide. COVID-19 is an infectious disease that can lead to severe acute respiratory syndrome. Under certain circumstances, the viral infection leads to excessive and uncontrolled inflammatory response, which is associated with the massive release of inflammatory cytokines in pulmonary alveolar structures. This phenomenon has been referred to as the “cytokine storm,” and it is closely linked to lung injury, acute respiratory syndrome and mortality. Unfortunately, there is currently no vaccine available to prevent the infection, and no effective treatment is available to reduce the mortality associated with the severe form of the disease. The cytokine storm associate with COVID-19 shows similarities with those observed in other pathologies such as sepsis, acute respiratory distress syndrome, acute lung injury and other viral infection including severe cases of influenza. However, the specific mechanisms that cause and modulate the cytokine storm in the different conditions remain to be determined. micro-RNAs are important regulators of gene expression, including key inflammatory cytokines involved in the massive recruitment of immune cells to the lungs such as IL1β, IL6, and TNFα. In recent years, it has been shown that nutraceutical agents can modulate the expression of miRs involved in the regulation of cytokines in various inflammatory diseases. Here we review the potential role of inflammatory-regulating-miRs in the cytokine storm associated with COVID-19, and propose that nutraceutical agents may represent a supportive therapeutic approach to modulate dysregulated miRs in this condition, providing benefits in severe respiratory diseases.

## Introduction: The COVID-19 Pandemic and the Need for Novel Therapeutic Approaches

It started in December 2019, in Wuhan, China, with a cluster of cases showing clinical symptoms of viral pneumonia. Because of this presentation and in reference to its origin, this disease was initially referred to as “Wuhan pneumonia” by the press. After sequencing its genome, researchers uncovered a novel coronavirus, which was temporarily named by the World Health Organization (WHO) the 2019 novel coronavirus (2019-nCoV). It is the seventh member of the coronavirus family to infect humans. This new infectious disease was officially named Coronavirus disease 2019 (COVID-19) on February 12, 2020 (Liu et al., 2020). COVID-19 rapidly spread throughout the world and on March 11, 2020, WHO formally recognized the situation as a pandemic (WHO, Covid-19 reports/20200311-rep-51). On August 5, 2020, 18,318,928 cases of COVID-19 have been confirmed, including 695,043 deaths reported to the WHO.

In severe cases, deaths from COVID-19 have been found to be related to Acute Respiratory disease Syndrome (ARDS). Therefore, the International Committee on Taxonomy of Viruses referred to COVID-19 as Severe Acute Respiratory Syndrome coronavirus 2 (SARS-CoV-2). COVID-19 associated ARDS is accompanied by an uncontrolled cytokine storm, which results from the systemic release of large amounts of pro-inflammatory cytokines and chemokines by immune effector cells. The immune system attacks the host violently, causing multiple organ failure which can lead to death (Li et al., 2020).

So far, no specific treatments or anti-virus vaccines are available to treat SARS-CoV-2 infection. There is an intensive mobilization of researchers all around the world to elucidate the pathogenic mechanisms of this viral infection in order to develop a cure for this disease. Meanwhile, to mitigate the impact of this virus on morbidity and mortality, one possible avenue is to mitigate the inflammatory response in order to prevent the cytokine storm. One possibility would be to use nutraceuticals. Indeed, nutraceuticals have been shown to boost the immune system and fight viruses with encapsulated RNA such as coronavirus (McCarty and DiNicolantonio, 2020). Some nutraceutical agents are effective in suppressing inflammatory pathways by acting on the modulation of specific microRNAs (miRs) (Quintanilha et al., 2017), small noncoding RNAs that can regulate gene expression by degrading mRNAs or by inhibiting RNA translation to proteins. Several studies have shown that dysregulation of miR expression can alter various biological processes such as inflammation (He and Hannon, 2004; Lee and Dutta, 2009; Sonkoly and Pivarcsi, 2009; Quintanilha et al., 2017). Moreover, dysregulation of miRs is now known to be involved in the development and the outcome of various diseases that can be associated with a cytokine storm including sepsis, ARDS, acute lung infection (ALI) and severe viral pneumonia (Quintanilha et al., 2017; Szilágyi et al., 2019; McCarty and DiNicolantonio, 2020). However, the role of miRs in the development of severe forms of COVID-19, as well as the potential use of nutraceuticals as therapeutic agents to modulate inflammatory miRs remain to be explored. In this article we review the role of miRs in the modulation of inflammatory factors involved in the cytokine storm in severe forms of COVID-19, and explore the potential effect of nutraceuticals as alternative therapeutic approaches in this context.

## Pathophysiological Aspects of COVID-19

### Characteristics of Coronavirus and Classical Players Involved in Cytokine Storm

Morphologically, coronaviruses (CoVs) are single-strand RNA viruses with spherical or multifaced particle shapes. They contain on their surface a spike protein that is required to adhere and infect the host cells. CoVs are the largest RNA viruses identified, with a genome that contains ∼13,000–16,000 base paired-end (Belouzard et al., 2012; Fehr and Perlman, 2015). Mechanistically, the positive viral RNA-strand serves as mRNA, allowing the production of its own replicase-transcriptase complex and also its structural proteins. Four main viral structural proteins called spike (S), envelope (E), membrane (M) and nucleocapsid (N) are found in CoVs (McBride et al., 2014). Additional viral accessory proteins are also important for the classification of CoVs. Seven CoVs members divided in two main types (α- or *β*-type) are able to infect humans with variations in terms of the efficiency of the infection, the specific clinical manifestations and the severity of the disease (McBride et al., 2014). The CoVs members associated with mild severity of upper respiratory tract infections include α-HCoV-229E, α-HCoV-NL63, α-HCoV-OC43 and *β* -HCoV-HKU1. More importantly, the members targeting the lower respiratory tract and associated with severe pathogenic acute respiratory distress syndrome (ARDS) potentially leading to fatal acute lung injury (ALI) include the *β*-SARS-CoV, *β* -MERS-CoV, and finally the *β* -2019-nCoV, also known as Covid-19 (van der Hoek et al., 2004; McBride et al., 2014; Jan et al., 2020; Wiersinga et al., 2020).

Over the past years, the cytokine storm has been identified as one of the common events found in critical acute pulmonary syndromes, but also as a key element in the pathogenicity and mortality related to ARDS/ALI following *β*-SARS-CoV and *β*-MERS-CoV infections (Gao et al., 2020; Tang et al., 2020). More recently, this cytokine storm has also been reported in severe forms of COVID-19 infection. In CoV infections, the cytokine storm is observed in the later stage of the infection, and is characterized by a rapid and uncontrolled release of several pro-inflammatory cytokines and chemokines by the infected lung tissue, leading to deregulated and excessive immune responses, critical inflammatory self-amplification circles, lung damage and in the more severe cases, mortality (Gao et al., 2020; Tang et al., 2020). Classically, several inflammatory factors are commonly involved in cytokine storm observed in *β*-SARS-CoV and *β*-MERS-CoV. Previous studies have shown that in the later stage of the infection, the expression level of the antiviral factor interferon (INF) is reduced in peripheral blood, lung epithelial cells, dendritic cells, and resident macrophages (Vabret et al., 2020). This is associated with a disproportionate increase in the production of the main pro-inflammatory cytokines IL-1β, IL-6 and TNFα, and the chemokines CCL-2, -3 and -5 (Mehta et al., 2020; Vabret et al., 2020; Ye et al., 2020). These factors lead to amplification of the inflammatory circle by promoting the recruitment in infected lungs of more monocytes, macrophages, and neutrophils which in turn can produce the same cytokines and chemokines, leading to excessive and sustained inflammatory tissue damage. In the serum of covid-19 patients with bilateral pneumonia and ARDS, recent studies have identified higher expression levels of IL-1β, IL-6, INF-y, IP-10, gm-CSF, TNFα, and MCP-1 (Johnson and Laloraya, 2020; Mehta et al., 2020; Ye et al., 2020). Importantly, IL-6 has been identified by researchers around the world as a key primary factor involved in Covid-19 cytokine storm, and it is also highly correlated to the pathogenic progression of ARDS (Grifoni et al., 2020; Magro, 2020; Zhang et al., 2020). Moreover, other studies have established a correlation between the serum cytokine levels, the degree of ALI and mortality rates. Together, these observations suggest that the extrapulmonary organ failures observed in severe forms of COVID-19 could at least partly be related to these excessive levels of cytokines that can recirculate in the peripheral blood and reach distant organs to induce tissue damage. We next focused to compiled differentially-analysis on patient with Covid-19 severe form to identify specific key inflammatory factors and immune cells, recently identified to drive the cytokine storm.

### Most Common Cytokines Identified in Severe Forms of Covid-19 Disease

The cytokine storm associated with severe forms of CoVs infections such as SARS and MERS represents a complex pathological condition involving serious pulmonary complication due to massive recruitment and infiltration of immune cells in pulmonary tissues (Gao et al., 2020; Ye et al., 2020). This can lead to epithelial and endothelial cell death, which in turn causes vascular leakage and ALI. In the case of covid-19 infection, specific biomarkers and mechanisms have recently been identified. For example, a strong upregulation of the proinflammatory cytokines TNF-α, IL-1β, and IL-6 was found in patients presenting severe forms of COVID-19 compared to patients with milder forms (Gao et al., 2020; Tang et al., 2020; Vabret et al., 2020). This observation suggests that these three cytokines act as key mediators of increased immune cell infiltration in the lung. These cytokines can be produced by a variety of cell types including B- and T-lymphocytes, monocytes and macrophages, dendritic cells as well as non-immune cells such as fibroblasts and endothelial cells (Sokol and Luster, 2015). Interestingly, IL-6 production is also induced by TNF-α and IL-1 acting as main activators (Confalone et al., 2010). Other signaling pathways including Toll-like receptor (TLR), prostaglandins, ROS and other cytokines can also trigger the production of IL-6 (Turner et al., 2014). Other proinflammatory factors such as IL-2, IL-7, MCP-1, GM-CSF and MIP-1a were reported to be increased in the peripheral blood of COVID-19 patients with severe forms of the diseases (Lingeswaran et al., 2020). This was also correlated with the severity of the disease. All of these factors are associated with immune cell recruitment that can amplify the acute and sustained inflammatory state in the lungs. Interestingly, other studies have observed an important lymphopenia characterized by a drastic decrease of CD4^+^ and CD8^+^ cells in patients with severe forms of COVID-19 (Tavakolpour et al., 2020). This was often associated with decreased circulating levels of IFN-γ, which is known to have antiviral properties by stimulating the recruitment of lymphocytes. In contrast, high leukocyte numbers and increased cytokine production have also been documented in patients with severe forms of COVID-19 (Lingeswaran et al., 2020; Mehta et al., 2020). However, in patients who died from the most severe form of COVID-19, it has been shown that monocytes and macrophages are the predominant immune cell types in lung infiltrates (Merad and Martin, 2020).

### microRNA: Small RNAs With Major Impact on Gene Expression

MicroRNA (miR or miRNA) are small non-coding single-stranded RNAs of 20–22 nucleotides that negatively regulate the expression of more than 60% of genes (Catalanotto et al., 2016). They act by degrading their target mRNA or by inhibiting its translation (Almeida et al., 2011). Due to their regulatory functions, they are involved in the majority of biological processes including development, growth, metabolism, survival, apoptosis, tissue repair, angiogenesis and, notably immune response and inflammatory processes (Lee and Dutta, 2009; Sonkoly and Pivarcsi, 2009; Almeida et al., 2011; Caporali and Emanueli, 2012). Over the past decade, advances in genomics and bioinformatic technologies have revolutionized the understanding of miR mechanisms of action. Notably, their roles as regulators of gene expression has been the subject of numerous studies (Giza et al., 2014). Historically, the first miR was discovered in 1993 by the Ambros group, who identified miR lin-4 in a *c. elegans* model (Lee et al., 1993; Almeida et al., 2011). However, it was not until a decade later that the physiological and pathological expression profile and function of specific miRs were investigated. In 2010, approximately 2,200 miRs in mammals and more than 1,000 miRs in humans were identified (Ardekani and Naeini, 2010). However, identification of new miRs seems to be constantly evolving since in 2015, such that to date approximately 2,500 miRs have been identified in humans (Friedländer et al., 2014). Importantly, it is now accepted that the epigenetic modulation of miR expression plays an important role in multiple pathological processes, including the immune response after viral infections.

### miR Biogenesis and Mechanism of Action

**FIGURE 1:**
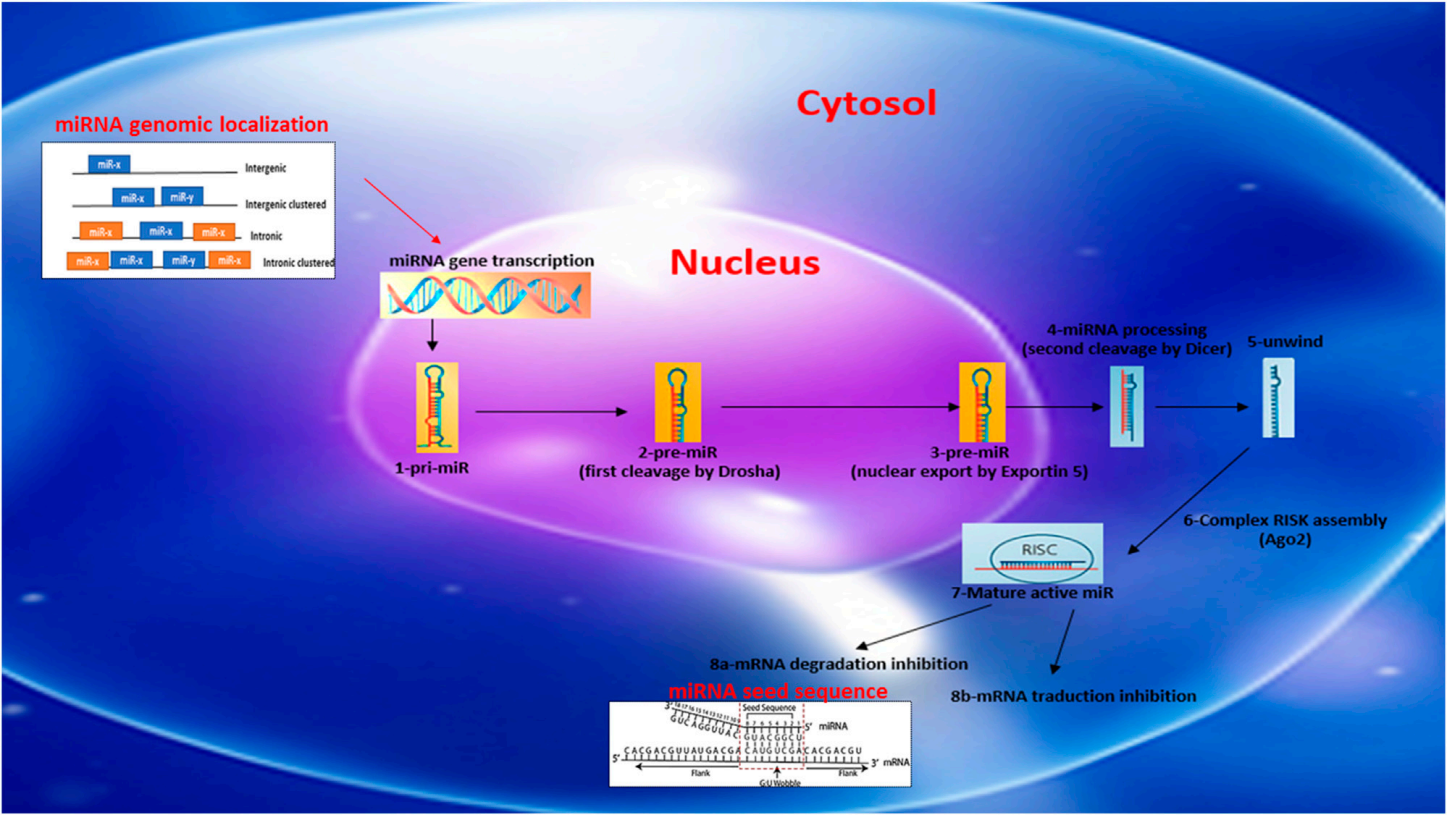
Schematic reprensentation of miRNA biosynthesis, chromosome localization nomenclature and miRNA/target seed sequence.

miRs are generally described as intergenic or intronic according to their genomic location (Figure 1). Intergenic miRs are transcribed by independent transcription units (TU). In the case of intronic miRs, they are transcribed at the same time as host genes generally coding for a protein (Ramalingam et al., 2014). miRs are synthesized autonomously from their own promoter sequences located between the genes–a process labeled as the canonical synthetic pathway (Figure 1). They can be transcribed alone or at the same time as other miRs located nearby, on the same “cluster” sharing a common promoter. The autonomous biogenesis of miRs by the canonical pathway begins with the transcription of a long primary transcript (pri-miR) of variable size and a stem-loop structure mediated by RNA polymerase 2. The pri-miR is then cleaved in the nucleus by an rnase complex (Drosha/DGCR8) to generate a precursor miR (pre-miR) of 70 nucleotides in length. The pre-miR is then exported from the nucleus to the cytoplasm by exportin-5 to undergo an enzymatic process. This second cleavage is mediated by the enzyme dicer and makes it possible to eliminate the stem-loop and to generate a mature miR duplex (double strand) of 22 nucleotides. The 5′ strand is called the guide strand, while the 3′ strand is called the passenger strand. Subsequently, one of the two strands will be incorporated into the RISK effector complex (RNA-induced silencing complex) with the help of a protein from the Argonaut family (Ago2). Ago2 also facilitates the binding of miRs to the target mRNA, leading to its subsequent repression. miRs recognize and bind their target mRNAs on the 3′UTR region using a specific sequence of eight nucleotides located on the 5′-UTR region of the miR called the “seed sequence” (Figure 1). The mechanisms of repression of target mRNAs are not fully defined. It is generally accepted that if the miR/mRNA pairing is perfectly complementary, the target mRNA will be degraded. In the case of an imperfect pairing, translation will be inhibited by various mechanisms. In both cases, the expression of the target gene will be downregulated. miRs generally have around 100 targets (mRNA) (Friedman et al., 2009; Friedländer et al., 2014; Giza et al., 2014; Ramalingam et al., 2014). The mature miRs and the pre-miRs can be localized in the cells or secreted by it in the circulation in a free form or sequestered in microvesicles (exoxome, apoptotic body, microparticle).

### Potential Role of miRs Related to Inflammatory Diseases and COVID-19 Cytokine Storm

In response to infection or injury, inflammation is the result of biological and pathophysiological cascades that involve both the innate and the adaptive immune systems. The alteration of this process can lead to abnormal, excessive, and sustained inflammation that will lead to tissue injury and in the most severe cases, tissue destruction (Chen et al., 2018). At the molecular level and in the early stage of the inflammatory process, the recruitment of immune cells is finely regulated by a well-known complicated network of pro- and anti-inflammatory factors such as cytokines and chemokines (Cicchese et al., 2018). However, the post-transcriptional mechanisms that are involved in the regulation of the expression level of cytokines and chemokines related to immune cell recruitment into inflammatory tissues is largely unknown. Notably, the role of miRs in COVID-19 cytokine storm remains to explored. As previously mentioned, each individual miR can act simultaneously on hundreds of different targets. Therefore one miR could fine-tune the modulation of several inflammatory signaling pathways at the same time (Sonkoly and Pivarcsi, 2009). Previous publications have reported alterations in the expression profile of miRs during inflammation in tissues, fluids and cell types (Mi et al., 2013). However, to date, no study has reported profiling of miRs in patients presenting severe forms of COVID-19. Accordingly, the following paragraphs will focus on clinical or pre-clinical studies describing examples of miR modulation during strong inflammatory responses such as sepsis, ARDS, ALI and severe pneumonias induced by the influenza virus.

As shown in Table 1, we have compiled miRs whose expression has been reported to be significantly altered in various diseases and inflammatory conditions, both in humans and in animal models of acute inflammation/infection (Table 1). For example, in the serum of patients with severe sepsis, the combined results of four clinical studies show that five miRs are strongly upregulated (miR-486, -182, -15b, -223 and -483), while 12 miRs are downregulated including miR-146 (Vasilescu et al., 2009; Wang et al., 2010; Wang et al., 2012; Tudor et al., 2014), a well-documented anti-inflammatory miR-146 that reduces the expression and activity of several pro-inflammatory mediators such IL1, TNFα, IRAK1, TRAF6 and NF-κB (Saba et al., 2014). In addition, several miRs have been found to be modulated (10 upregulated and seven downregulated) in two preclinical studies of severe pulmonary inflammation in animal models of ARDS and ALI, two conditions that resemble the lung pathology found in patients with severe forms of COVID-19 (Huang et al., 2014; Zhu et al., 2017). Interestingly, miR-127 was found to be significantly increased in these conditions. miR-127 is a well-known pro-inflammatory miRNA that induces macrophage M1 phenotype trough the induction of IL-6, TNFα, IL-1β, and by decreasing the anti-inflammatory cytokine IL-10 (Ying et al., 2015). Interestingly, in a study of influenza virus-induced lung inflammation, a cytokine storm-mediated COVID-19-like situation, the intracellular expression of several miRs were modulated in lung cells A549 infected with influenza, suggesting that altered local expression of miRs in tissue can also influence the subsequent local inflammatory response (Li et al., 2010; Huang et al., 2014). Among the miRs found to be increased in inflammatory conditions, two members of the Let-7 family (Let-7f and Let-7a) known for their dual pro-inflammatory/pro-angiogenic functions have been reported to be induced by the influenza virus (Wang and Olson, 2009; Teng et al., 2013). Interestingly, alterations of miR expression have also been documented in children with Respiratory Syncytial Virus Infection (CRSVI) (Inchley et al., 2015) and in patients with severe bi-lateral pneumonia (Huang et al., 2019), which is directly linked to mortality in COVID-19 patients on mechanical ventilation (see Table 1).

**TABLE 1:** Modulation of miRNA expression in diverse inflammatory diseases, including clinical and pre-clinical studies.

Inflammatory diseases	Human or models	Upregulated miRNAs	Downregulated miRNAs	References
Sepsis (4 clinical studies included)	Human patients with sepsis	miR-486, miR-182, miR-15b, miR-223, miR-483-5p	miR-150, miR-342-5p, miR-223, miR-146b, miR-223, miR-146a, miR-122, miR-193b, miR-499-5p, miR-23a, miR-26a, miR-342	Vasilescu et al. (2009); Wang et al. (2010); Wang et al. (2012); Tudor et al. (2014)
Acute respiratory distress syndrome (ARDS) and acute lung injury (ALI) (2 animal studies)	Rat model of ARDS and ALI	miR-181, miR-92a, miR-424miR-344, miR-346, miR-99a, miR-127, miR-128b, miR-135b, and miR-30a/b	miR-24, miR-26a, miR-126, and Let-7a, b, c, f	Wang et al. (2010); Zhu et al. (2017)
Lung inflammation related to influenza virus	Mice lung and human A549 lung cells infected with influenza	let7a, let7f, miR-145, miR-21, miR-223, miR-101, miR-193, miR-23b, miR-30e	miR-29, miR-200, miR-193, miR-27, miR-29, miR-30	Li et al. (2010); Huang et al. (2014)
Children respiratory syncytial virus infection (CRSVI)	Human patients with CRSVI	miR-155, miR-31, miR-203a, miR-16 and let-7days	miR-34b, miR-34c, miR-125b, miR-29c, mir125a, miR-429 and miR-27b	Inchley et al. (2015)
Severe pneumonia	Human patients with severe pneumonia	miR-450a-5p-miR-103a-3p and miR-103b-5p-miR-98-5p	N/A	Huang et al. (2019)

In order to illustrate the importance of miRs in the regulation of inflammation, we have compiled miRs whose function is known to affect the expression of key inflammatory mediators, and that could therefore potentially be involved in the cytokine storm associated with COVID-19 (Table 2). For example, miR-125 is known for its anti-inflammatory properties by reducing TLR/NF-κB activities related to myelodysplastic syndromes in Meg-01 and K562 cells, but it is also negatively correlated to CRP, IL-17 and TNFα levels in the plasma of patients with Crohn disease (Gañán-Gómez et al., 2014; Sun et al., 2017). miR-125a may act as a NF-κB inhibitor upon TLR stimulation, and its effects may therefore depend on the status of the TLR pathway. In addition, miR-146, which is strongly reduced in sepsis (Table 1), is well known for its anti-inflammatory properties by negatively regulating NF-kB activities and by directly targeting IRAK-1 and TRAF6 (Taganov et al., 2006). miR-146 also plays an anti-inflammatory function in human bronchial epithelial cells during asthma (Lambert et al., 2018). Taken together, these findings indicate that miR-146b repress IRAK-1 and TRAF6 signaling and suggest that it could be induced in certain inflammatory conditions as a feedback mechanism to limit inflammation. In alveolar macrophages during ARDS, miR-199 is known for its pro-inflammatory action contributing to the induction and release of key inflammatory cytokines such as TNFα, IL1β and IL6 (Li et al., 2018). As previously mentioned, IL-6 has been identified as a key factor positively correlated with severe forms of COVID-19. Decreased expression of miR-199a with antagomir199 has previously been shown to play a protective role in mouse alveolar macrophage during ARDS through decreased expression of TNFα, IL1β and IL6. In addition, other miRs such as miR-124 and miR-223 have anti-inflammatory properties in macrophages stimulated with LPS. miR-124 decreases TNFα and IL-6 through STAT3 and TACE pathway (Sun et al., 2013), while miR-223 decreases IL6 and IL1β via TLR inhibition (Chen et al., 2012). In summary, this section demonstrates that several miRs with pro- and anti-inflammatory properties are altered in inflammatory conditions, both systemically (plasma/serum) and locally in lung tissues and cells. The next section will document the potential therapeutic impact of modulating inflammatory miRs using nutraceutical agents.

**TABLE 2:** Examples of miRNAs regulating inflammatory processes related to cytokine/chemokine production and immune cells recruitment.

miRNAs ID	Inflammatory process involved	Model/disease and tissues or cells lines	Overall potential effects of miR modulation	References
miR-125	Anti-inflammatory properties by reducing TLR/NF-κB activities negatively correlated with CRP and CDAI, IL-17, TNF-a production	Myelodysplastic Syndrome/Meg-01 and K562 Crohn'|’s disease/human plasma	miR-125a may act as NF-κB inhibitor upon TLR stimulation. These results indicate that miR-125a is involved in the fine-tuning of NF-κB activity and that its effects may depend on the status of the TLR pathway reduced expression of miR-125 could lead to upregulation of CRP et CDAI, IL-17, TNF-a production	Gañán-Gómez et al. (2014); Sun et al., (2017)
miR-146a	Anti-inflammatory properties by negatively regulating NF-kB activities by directly targeting IRAK-1 and TRAF6 pulmonary anti-inflammatory effects	Human acute monocytic leukemia cell line THP-1 asthma/human bronchial epithelial cells	miR-146a repress IRAK-1 and TRAF6 signaling. miR-146a is potentially induced in inflammatory conditions as a feedback mechanism to limit inflammation	Taganov et al. (2006); Lambert et al. (2018)
miR-142, -101, -29a, -29c, and -141-3p	Pro-inflammatory effects; these miRs are upregulated in sepsis and positively correlated with IL-6 and TNF-α level in blood	Still disease from sepsis/serum	Could potentially act by targeting negative regulator of IL-6 and TNF-α inducer	Hu et al. (2018)
miR-127	Pro-inflammatory effects regulating the polarization of macrophage M1 vs M2 (miR-127 expression activates the M1 phenotype)	Murine lung inflammation injury model induced by LPS	miR-127 activates the M1 phenotype by inducing the expression of IL-6, TNFα, IL-1β, and decreasing the anti-inflammatory cytokine IL-10	Ying et al. (2015)
miR-124 miR-223	Anti-inflammatory properties	Murine macrophage stimulated with LPS	miR-124 decreases TNFα and IL-6 through STAT3 et TACE pathway miR-223 decreases IL-6 and IL1β via TLR inhibition	Chen et al. (2012); Sun et al. (2013)
miR-199	Pro-inflammatory action	ARDS/Mice alveolar macrophage	Decreased expression of miR-199a with antagomir199 plays a protective role in mouse alveolar macrophage ARDS through decreased expression of TNFα, IL1β, IL6	Liu et al. (2018)

## Nutraceutical Agents Modulating the Expression of Inflammation-Modulating miRs

“Nutraceutical” is a broad term used to describe any natural compound derived from food sources, herbal products or dietary supplements. The term combines the words “nutrient” (a food component) and “pharmaceutical” (a medical drug) and was invented by Dr. Stephen DeFelice in 1989 (Kalra, 2003). In addition to their basic nutritional value, nutraceutical agents have been shown to exert physiological benefits or provide health protection against different diseases. Indeed, it was recently reported that certain nutraceuticals may boost the immune system and help fight viruses with encapsulated RNA such as the influenza and coronavirus (McCarty and DiNicolantonio, 2020). Several pre-clinical and clinical studies have shown that nutraceutical agents are particularly effective in suppressing inflammatory pathways (Moss and Ramji, 2016; Gupta et al., 2018). Among the possible mechanisms of action of these natural compounds is their ability to modulate the expression and the activity of a number of microRNAs. In this section, we summarize studies that have demonstrated the potential effect of certain nutraceutical agents on miRNAs, especially those that regulate key inflammatory factors related to COVID-19-associated cytokine storm (Quintanilha et al., 2017; Sabino et al., 2019).

### Nutraceutic Targeting of miRs Involved in the Modulation of COVID-19- Associated Cytokine Storm

#### Resveratrol

Resveratrol (*trans*-3,5,4′-trihydroxystilbene) (RSV), a polyphenolic compound, is found in red grapes, berries, peanuts and bamboo. It is most abundant in the skin of grapes and is principally consumed in the form of grape derived red wine. Several studies have shown that RSV has anti-inflammatory, antioxidant, anti-carcinogenic, anti-aging properties, and cardio-protective effects *in vivo* and *in vitro* (Bao et al., 2012; McCubrey et al., 2017; Alharris et al., 2018). However, it is difficult to establish the precise effective dose related to the beneficial anti-inflammatory effects in humans because studies have used a large range of doses (150 mg–2.5 g per day). Concentrated extracts of resveratrol have been used in order to overcome the difficulty of obtaining this concentration from the diet (Ramírez-Garza et al., 2018). For instance the amount of resveratrol in a 150 ml glass of red wine is 0.015–2.15 mg, while small fruits such as blueberries, raspberries and cranberries contain between 0.6 and 2.4 mg (Chachay et al., 2011). The recommended amount of RSV in the diet is around 12.5 mg/kg body weight. These concentrations are obtained by rough extrapolations from animal experiments, most of which required daily dosages of 5–100 mg/kg body weight to reach a specific biological effect. Several experts claim that a daily dosage of 1 g of resveratrol is effective for the treatment of diverse disorders in humans. It is not possible to ingest up to 1 g of resveratrol/day by consuming conventional food products. Alternatives that are offered by many companies include a variety of (sometimes outrageously expensive) nutritional supplements with precisely defined resveratrol content (Weiskirchen and Weiskirchen, 2016). It has been shown that RSV also exhibits antiviral properties against a variety of viral pathogens. In particular, RSV at a concentration of 125–250 µM was shown to suppress the replication of the Middle East Respiratory Syndrome Coronavirus (MERS-CoV) in Vero E6 cells *in vitro* (Lin et al., 2017; Marinella, 2020). RSV may be a promising molecule since no adverse effect was observed *in vivo*, even when high doses were administered to animals for prolonged periods of time. RSV was or is currently being tested in over 110 clinical trials (Latruffe et al., 2015; McCubrey et al., 2017; Marinella, 2020). Although the molecular mechanisms related to the pleiotropic activities of RSV are still largely unknown, a few studies have investigated the potential role of miRNAs in resveratrol-mediated anti-inflammatory effects (Alharris et al., 2018). In lipopolysaccharide (LPS)-stimulated human THP-1 macrophages, a previous study found that RSV used at a concentration of 25–200 µM (with maximal peak effect at 25 µM) markedly increases the expression of miR-Let-7A (Song, et al., 2016), which attenuates the expression of the pro-inflammatory cytokines TNF-α and IL-6 (see Table 3). Also, in LPS-induced inflammatory injury in PC-12 neuroblastic cells, it was reported that RSV at a concentration of 10–25 µM can decrease IL1β, IL6, and TNFα expression through upregulation of miR-132. This effect of RSV improves cell viability and reduces apoptosis by suppressing the inflammatory response (Zhang et al., 2019). Both miR-132 and miR-Let-7A have been shown to be important for the modulation of the inflammatory response, and for cell survival/apoptosis. RSV was reported to be a promising oral supplement for the treatment of osteoarthritis (Gu et al., 2016). The levels of IL-1β, IL-6 and TNF-α were significantly reduced by a treatment of RSV (30 µM) in LPS-induced inflammatory injury of ATDC5 cells. RSV thus seems to act by upregulating miR-146b (Jin et al., 2018). Similarly, RSV significantly inhibited LPS-induced generation of TNFα, IL1β and IL6 in BV2 mouse microglial cells via upregulation of miR-146a-5p (Ge et al., 2019). The cells were treated with RSV (3, 10, and 30 µM) 24 h prior to LPS treatment for 6 h. Pretreatment with RSV at the concentration of 3 µM showed no effect on LPS-induced upregulation of cytokines. However, RSV (10 and 30 µM) pre-exposure significantly attenuated LPS-induced mRNAs increase of TNF-a by 54.6 and 77.9%, IL-1b by 38.9 and 65.9%, IL-6 by 21.7 and 43.6%, respectively. Pretreatment with 10 and 30 µM of RSV 24 h before LPS exposure resulted in significantly increased levels of the anti-inflammatory miR-146a-5p in LPS treated cells (5.21-fold vs. LPS at 10 μM; 8.41-fold vs. LPS at 30 µM). MiR-146b as well as miR-146a-5p are important regulators of innate immune responses. Through the modulation of different miRNAs, *in vitro* studies suggest that RSV (25–250 µM) mediates anti-inflammatory effects by targeting the three main cytokines commonly involved in severe forms of COVID-19.

**TABLE 3:** Neutraceutical targeting of miRs that potentially attenuate inflammatory cell recruitment.

Nutraceutical agent	miR modulated	Modulation of Expression	Cell/sample/tissue	Pathology/disease/model	Predicted potential impact on acute lung injury mediated by cytokine strom	References
Resveratrol	miR-let7a	Upregulated	THP-1 cells	LPS-stimulated macrophages	Decreased expression of TNF-α and IL-6	Song et al. (2016)
	miR-132	Upregulated	PC-12 cells	LPS-induced inflammatory injury of PC-12 neuroblastic cells	Decreased IL-1β, IL-6, and TNF-α expression	Zhang et al. (2019)
	miR-146b	Upregulated	Mouse chondrogenic cell line ATDC5	LPS-induced inflammatory injury of ATDC5 cells	Decreased IL-1β, IL-6, and TNF-α expression	Jin et al. (2018)
	miR-146a-5p	Upregulated	BV2 mouse microglial cells	LPS-induced inflammatory injury of BV2 cells	Decreased mRNA levels of TNF-α, IL-1β, and, IL-6	(Ge, Zhong et al., 2019)
Quercetin	miR-221	Downregulated	WI-38 lung fibroblasts	LPS-caused inflammatory damage of WI-38 lung fibroblasts	Decreased mRNA levels and expression of TNF-α and IL-6	(Wang et al., 2019)
	miR-369-3p	Upregulated	Bone marrow derived dendritic cells (BMDCs)	LPS-stimulated BMDCs	Decreased mRNA levels and expression of TNF-α and IL-6	(Galleggiante et al., 2019)
	miR-124	Upregulated	Human kidney-2 cells	LPS-induced inflammatory injury of HK-2 cells	Decreased expression of TNF-α and IL-6	Guo et al. (2020)
Vitamin D	miR-125b	Downregulated	THP-1 cells and in lamina propria mononuclear cells (LPMC)	LPS-stimulated macrophages and DSS-induced colitis mice	Decreased expression of TNF-α and IL-6	Zhu et al. (2019)
	miR-155	Downregulated	Raw264.7 cells and serum	LPS-treated macrophages and LPS-induced sepsis mice in WT and miR155 KO mice	Decreased expression of TNF-α and IL-6	Chen et al. (2013)
Curcumin	miR-155	Downregulated	Raw264.7 and THP-1 cells; liver and kidney tissues	LPS-treated macrophages LPS-induced sepsis in mice	Decreased expression of TNF-α and IL-6	Ma et al. (2017)
	miR-199b-5p	Upregulated	BV2 microglial cells	LPS-induced BV2 cells	Decreased expression of TNF-α and IL-1β	Gao et al. (2019)
Ginseng	miR-132	Upregulated	Lung fibroblast MRC-5 cells	LPS-induced MRC-5 cell injury	Decreased IL-1β, IL-6, and TNF-α expression	Cong et al. (2019)
	miR-181a	Upregulated	WI-38 lung fibroblasts	LPS-caused inflammatory damage of WI-38 lung fibroblasts	Decreased mRNA levels and expression of IL-1β, TNF-α and IL-6	Qian et al. (2019)
	miR-26a	Upregulated	Human renal proximal tubular epithelial cells and human kidney-2 cells	LPS-treated human RPTECs and HK-2 cells	Decreased mRNA levels and expression of IL-1β, TNF-α and IL-6	Liu et al. (2019)
Green tea polyphenols	miR-9	Upregulated	Mouse chondrocytic ATDC5 cells	LPS-induced osteoarthritis in ATDC5 cells	Decreased expression of TNF-α, IL-6 and MCP-1	Zhang et al. (2019)

#### Quercetin

Quercetin (3, 3′, 4′, 5, 7-pentahydroxyflvanone) is part of the flavonoid family. Due to its high abundance (4–79 mg/100 g) in many fruits and vegetables including apples, grapes, onions, artichoke/fennel/celery, beans, apricots, plums, turnips, peppers, strawberries, tomatoes and broccoli, quercetin can be consumed in appreciable amounts, with an average daily absorption via the diet estimated at 50–800 mg (Boesch-Saadatmandi et al., 2012; Li et al., 2016; Kocic et al., 2019). Other studies found that in determined food, the highest concentration is 234 mg/100 g of edible portion in capers (raw), the lowest concentration is 2 mg/100 g of edible portion in black or green tea (Camellia sinensis), and the estimated absorption is from 3% to 17% in healthy individuals receiving 100 mg of quercetin. The relatively low bioavailability of quercetin may be attributed to its low absorption, extensive metabolism and/or rapid elimination (Li et al., 2016). Among its pharmacological properties, quercetin has anti-inflammatory effects, antiproliferative effects on tumor cells, and cytoprotective effects against oxidative stress. As mentioned above for resveratrol, the effective dose of quercetin supplementation is highly variable in animal and human studies. For example, in a rat arthritis model, a daily dose of 80 mg/kg, inhibited both acute and chronic inflammatory phases (Guardia et al., 2001). In humans, in a 12 weeks clinical trial, quercetin at a dose of 1,000 mg/day exerted anti-inflammatory properties characterized by a reduction of the upper respiratory tract infection rates in middle and older age subjects (Heinz et al., 2010). In addition, an intake of 500 mg of quercetin significantly decreased inflammatory markers such as TNF-α and IL-6 in women with type 2 diabetes, a risk factor for CVD (Zahedi et al., 2013). *In vitro,* the effective anti-inflammatory concentration of quercetin to inhibit IL-1 and TNFa expression is between 10 and 100 µM in N9 microglial cells (Bureau et al., 2008). It can potentially boost natural immunity and contribute to prevent a number of chronic diseases (Galleggiante et al., 2019). A few studies have investigated the modulation of miRNAs by quercetin in the context of inflammation. Another group (Wang et al., 2019) showed that downregulation of miRNA-221 by quercetin in the setting of LPS-induced inflammatory damage leads to decreased mRNA and protein expression levels of IL6 and TNFα in WI-38 lung fibroblasts. miR-221 is known to activate factors related to inflammation such as NF-κB and c-Jun N-terminal kinase (JNK) (Zhao et al., 2016; Qian et al., 2017). Another study found that treatment of LPS-stimulated bone marrow derived dendritic cells (BMDCs) with quercetin significantly increases miR-369-3p expression levels. This upregulation of miR-369-3p leads to decreased mRNA and protein levels of TNFα and IL6 (Galleggiante et al., 2019). A recent study also showed that quercetin, by up-regulating miR-124 in LPS-induced inflammatory injury of human kidney-2 cells (HK-2 cells), can reduce generation of IL6 and TNFα (Guo et al., 2019).

#### Vitamin D

Vitamin D, a fat-soluble vitamin, exists in two forms: D_2_, which comes from vegetable origin and D_3_, which is mainly synthesized in the skin when a cholesterol precursor, 7-dehydroxycholesterol, is exposed to UVB radiation. Dietary sources of vitamin D include oily fish such as cod liver oil (210 µg/100 g) and salmon (5–13 µg/100 g), mushrooms (13–30 µg/100 g) and foods fortified with vitamin D like dairy products (O'Mahony et al., 2011). There is much debate in the literature as to which cut-offs to use for defining vitamin D deficiency. However, recent studies suggested serum concentrations of 75 nmol/L or higher as adequate levels, with values below this level indicating deficiency (O'Mahony et al., 2011). Vitamin D_3_ or oral vitamin D is first converted to 25(OH)D in the liver, then to its active form, 1,25-Dihydroxyvitamin D (1,25(OH)_2_D_3_) (calcitriol), in the kidneys or sometimes in other organs and in cells of the immune system. In fact, in these cells, 1,25(OH)_2_D_3_ plays an important role in the control of inflammatory responses (Carvalho et al., 2017; Grant et al., 2020; Zabetakis et al., 2020).

The precise dose of vitamin D supplementation that exerts anti-inflammatory effects in humans remained to be determined. A previous study showed that a consummation of 500–5,000 IU of vitamin D3 per day exerts a dose-dependent anti-inflammatory effect on gingivitis in patients (Hiremath et al., 2013). A meta-analysis combining seven clinical studies concluded that vitamin D-supplemented in patients leads to lower plasmatic concentrations of TNFa compared to controls (n = 380; *p* = 0.04). However, no effects were observed on the levels of C-reactive protein, IL-10 or IL-6. These findings suggest that vitamin D supplementation may have specific, but modest effects on inflammatory markers (Rodriguez et al., 2018). Recent animal and *in vitro* studies have investigated the anti-inflammatory effects of vitamin D through the modulation on miRNAs. Zhu X et al. observed that in LPS-stimulated THP-1 cells, as well as in lamina propria mononuclear cells (LPMCs) isolated from DSS-induced colitis mice, the downregulation of miR-125b expression by 1,25(OH)_2_D_3_ induces M1 macrophage polarization toward the M2 anti-inflammatory subtype. As a consequence, TNF-α and IL-6 expression are decreased (Curtale et al., 2019). Another study showed *in vivo* that 1,25(OH)_2_D_3_ supplementation (200 ng/kg per day for one week) downregulates miR-155, TNFα and IL6 in LPS-treated mouse macrophage RAW264.7 (Chen et al., 2013) and the specificity of the effect was validated by comparing wild type mice to miR-155-null (KO) mice after intraperitoneal LPS challenge. Since respiratory tract infections cause an increase in production of pro-inflammatory cytokines and chemokines, 1,25(OH)_2_D_3_, may participate in attenuating the susceptibility to acute (severe) viral-induced respiratory distress syndrome, like COVID-19.

#### Curcumin

Curcumin [1,7-bis(4-hydroxy-3-methoxyphenyl)-1,6-heptadiene-3,5-dione] is a natural polyphenol extracted from the rhizomes of the plant *Curcuma longa*. It is the bioactive ingredient of turmeric and it is also found in other plants like ginger. Curcumin is commonly used as a spice, being responsible for the yellow pigmentation of the curry (Bao et al., 2012; Hewlings and Kalman, 2017; McCubrey et al., 2017). This bioactive dietary compound exerts beneficial potential protective effects against various diseases including virus infections and cancers, particularly those associated with inflammation and interlinked oxidative stress (Quintanilha et al., 2017; Zahedipour et al., 2020). Animal studies have shown that curcumin is rapidly metabolized, conjugated in the liver, and excreted in the feces, therefore having limited systemic bioavailability suggesting that high doses are required to reach beneficial anti-inflammatory effects. For example, a 40 mg/kg intravenous dose of curcumin in rats resulted in complete plasma clearance after 1-h. An oral dose of 500 mg/kg in rats resulted in a peak plasma concentration of only 1.8 ng/ml, with the major metabolites identified being curcumin sulfate and curcumin glucuronide. The dose used in humans in clinical studies (mostly in cancer patients) varies between 0.5 and 10 g per day administered orally (Jurenka, 2009). Here we review studies describing a link between miRNAs and the modulating effect of curcumin on the inflammatory response.

Ma et al. showed *in vitro* that curcumin exerts dose dependent (5–15 µM) anti-inflammatory effects through miR-155, leading to decreased secretion of TNFα and IL6 in LPS-stimulated mouse macrophages RAW264.7, and in human monocyte THP-1 macrophages cells. In a mouse model of LPS-induced sepsis *in vivo*, these authors found that 20 mg/kg of curcumin treatment for 3 days before LPS intraperitoneal injection suppresses the inflammatory response in liver and kidney by inhibiting the cytokines TNFa and IL-6 through targeting miR-155 (Ma et al., 2017). miR-155 has emerged as a key regulator of immune functions and a factor involved in the development of inflammation-related diseases (Testa et al., 2017). *In vitro*, curcumin (2–8 µM) was recently found to diminish the expression of TNFα and IL1β in LPS-treated BV2 microglial cells, by increasing the level of miR-199b-5p (Gao et al., 2019). Although miR-199b-5p is a well-documented tumor suppressor, it was also recently proposed as a potential biomarker of sepsis and septic shock (Reithmair et al., 2017). Therefore, since curcumin has been shown to strongly inhibit several pro-inflammatory cytokines, particularly those implicated in the cytokine storm, and considering its antiviral activities, this nutraceutical compound may be a promising candidate for the management of severe coronavirus infections.

#### Ginseng

Ginseng has been used for a long time as a health supplement and a traditional herbal medicine. It is extracted from the root of a *Panax ginseng*, one of the 13 species of perennial plant of genus Panax, family of Araliaceae, cultivated in Eastern Asia and North America. Studies on the pharmacological and medicinal effects of ginseng have focused on ginsenosides, which are the main bioactive compounds in ginseng. Ginsenosides are steroid-like saponins and numerous types of ginsenosides have been reported to have physiological effects in a number of human illnesses such as cancers, diabetes, as well as neuronal, cardiovascular and inflammatory diseases (Paik et al., 2019; Yu et al., 2019). To date, more than 150 ginsenosides have been isolated from ginseng, 40 of which have been found in Panax ginseng in very low and variable quantity, therefore needing to be concentrated in therapeutic studies in order to reach effective dosing (Liu et al., 2019). Pharmacokinetic studies of selected ginsenosides in rats, dogs or human plasma, have shown that supplementation with different ginsenosides at doses of 10–300 mg/kg resulted in variable bioactivity (1–64%) depending on the compound and the model, highlighting the complexity of extrapolating these results to humans (Lü et al., 2009). In a study investigating whether Korean red ginseng (KRG) can play a role in repressing the development of chronic nonbacterial prostatitis (CNP) in male Wistar rats, KRG injected subcutaneously at doses of 0.25 and 0.5 mg/kg significantly decreased the expression of key pro-inflammatory cytokines such as IL-6, IL-1, TNFα and COX2 (Tabolacci et al., 2019). *In vitro*, a study evaluated the anti-inflammatory effects of ginsenoside Rg1 against LPS-induced microglia activation in BV2 microglial cells and ventral mesencephalic primary microglial culture, and concluded that a concentration of 10 µM of Rg1 could attenuate LPS-induced upregulation of TNFa, IL-1b, iNOS, COX-2 mRNA and protein levels (Gao et al., 2019). It has recently been reported that various ginsenosides can directly modulate miRNAs, providing an alternative pathway through which ginseng can exert its anti-inflammatory function (Dong et al., 2019). miRNA-132 is upregulated by notoginsenoside R1 (NGR1), the main active component extracted from the roots of Panax notoginseng (Burk.), in a model of cell injury involving LPS-treated lung fibroblast MRC-5 (Cong et al., 2019). miRNA-132 upregulation leads to decreased expression of the inflammatory cytokines IL1β, IL6 and TNFα (Salvi et al., 2019). Similarly, NGR1was also found to upregulate miR-181a in LPS-induced inflammatory damaged WI-38 lung fibroblasts, leading to decreased mRNA and protein expression of IL1β, IL6 and TNFα (Qian et al., 2019). Pro-inflammatory regulatory miR26a was also found to augment in response to NGR1 in LPS-treated human kidney-2 cells (HK-2 cells) and human renal proximal tubular epithelial cells (RPTECs); this resulted in reduced release of IL1β, IL6 and TNFα (Liu et al., 2019).

#### Green Tea Polyphenols and Other Compounds

Green tea is extracted from the leaves and buds of the plant *Camellia sinensis*. Because of its many health benefits, green tea is one of the most widely consumed beverages worldwide. Green tea polyphenols (GTP) are the active compounds in green tea and they are also commonly known as catechins. The main ones are epigallocathechin gallate (EGCG), epicathechin gallate, epichatechins and flavanols, which are responsible for many of the biological activities of green tea including the antioxidant, anti-inflammatory, anti-angiogenesis, antidiabetic and anticarcinogenic effects (Otton et al., 2018; Torres et al., 2019; Yan et al., 2020). A 250 ml cup of brewed green tea provides approximately 25–60 mg of EGCG (roughly 50–60% of the catechins are EGCG). *In vitro*, a study in human gingival epithelial keratinocytes (HGEK) treated with LPS showed that green tea extracts (2.5, 5, and 10 mg/ml) decreased IL-β1, IL-6 and TNFα gene expression by more than 10-fold (Hagiu et al., 2020). Another study in rats with acetic acid-induced colitis demonstrated that EGCG (50 mg/kg/day for seven days) exerts anti-inflammatory activity by inhibiting the production of TNFa, IFN-gamma and NF-kappaBp65 (Ran et al., 2008). Importantly, Zhang Q et al. have shown that GTP can directly modulate the expression of miRNAs implicated in the control of inflammation. They observed in a model of LPS-induced inflammation in mouse chondrocyte ATDC5 cells that GTP increases miRNA-9, which suppresses pro-inflammatory cytokines and chemokines (Zhang et al., 2019). It would thus be tempting to speculate efficacy of GTP in severe COVID-19 infections.

Several other nutraceutical compounds could also help modulating inflammation in the context of COVID-19. For example the potential therapeutic effect of lactoferrin, a non-toxic pleiotropic glycoprotein, has been studied in several viruses including severe acute respiratory syndrome coronavirus (SARS-CoV), which is closely related to the novel severe acute respiratory syndrome coronavirus 2 (SARS-CoV-2) causing COVID-19. In fact, lactoferrin exhibits several interesting immunomodulatory and anti-inflammatory characteristics that could positively modify the host response to infections. However, additional studies are needed to specifically define the modulating effect of lactoferrin on the expression of miRs, especially in the context of severe inflammatory diseases (Chang et al., 2020).

## Conclusion

ARDS is by far the main cause of mortality associated with severe coronavirus infections such as MERS, SARS-CoV-1 and SARS-CoV-2 (COVID-19), for which there are no pharmacological treatments to date. Management of ARDS remains largely supportive using different ventilatory modalities. The rapidly progressive nature of respiratory failure corresponds with a cytokine storm, including IL1β, IL6, IL8, TNFα, detected in broncho-alveolar lavage fluids (Butt et al., 2016; Felsenstein et al., 2020; Schett et al., 2020). Concordantly, IL6 promoter variant is associated with reduced severity of ARDS (Martín-Loeches et al., 2012), likewise, IL-1 receptor antagonist-null mice have delayed resolution of lung inflammation in ARDS (Hudock et al., 2012). Attempts to treat patients using approved anti-IL-1 and IL-6 have been somewhat successful in ARDS (Shakoory et al., 2016; Toniati et al., 2020), albeit there are concerns with consequent risks of immunosuppression using currently available anti-IL-1 and anti-IL-6 (respectively, Kineret and Tocilizumab (Felsenstein et al., 2020; Zhang et al., 2020). Concomitant down-regulation of numerous (potentially detrimental) pro-inflammatory cytokines lends itself to better solutions to treat ARDS in COVID-19. Accordingly, safe modulators of miRs would be well-suited for this purpose. In this context, nutraceuticals (administered individually or in combination) such as the ones presented in this review (RSV, quercetin, vitamin D, curcumin, ginseng, GTP), that exert anti-inflammatory properties by affecting key miRs, may be plausible alternatives to single target therapeutics to effectively attenuate the severity of ARDS associated with COVID-19 and the likes, and improve outcome.

## Author Contributions

MD conceptualized and designed the study. MD, MW, IL, PR carried out the literature research of the review. MD prepared the initial draft of the manuscript and Figure 1. AR and SC revised the manuscript and figures. PH, AR, SC provided expert advice and recommendations. All authors read and approved the final manuscript.

## Funding

MD is a recipient of a post-doctoral fellowship award from Hopital Maisonneuve-Rosemont and from the Fonds de Recherche en Ophtalmologie de l'Universite de Montreal. AR holds a grant from the Canadian Institute of Q17 Health Research (CIHR MOP-123490) and the Heart and Stroke Foundation of Canada (HSFC G-17-0019106). SC holds a Canada Research Chair (Vision Science) and the Leopoldine Wolfe Chair in translational research in age-related macular degeneration. The study was financed by grants from Canadian Institutes of Health Research (grant number—MOP12532), March of Dimes Birth Defects Foundation, Fonds de la Recherche du Québec—Santé (FRQS)/Québec Vision Health Network (grantee: SC).

## Conflict of Interest

The authors declare that the research was conducted in the absence of any commercial or financial relationships that could be construed as a potential conflict of interest.
